# The Arabic Leary Impostorism Scale: initial psychometric evaluation in a university student population

**DOI:** 10.3389/fpsyg.2026.1973835

**Published:** 2026-10-21

**Authors:** Nasser M. AbuDujain, Ahmed Alswailem, Saif K. Alotaibi, Mohammed Hamad Alhumud, Joud S. Almutairi, Haytham I. AlSaif, Safaa A. Hakami, Max P. Jansen, Turky H. Almigbal

**Affiliations:** 1 Department of Family and Community Medicine, College of Medicine, King Saud University, Riyadh, Saudi Arabia; 2 College of Medicine, King Saud University, Riyadh, Saudi Arabia; 3 School of Public Health, Faculty of Medicine, Imperial College London, London, United Kingdom; 4 University Medical Center, King Abdullah bin Abdulaziz University Hospital, Princess Nourah bint Abdulrahman University, Riyadh, Saudi Arabia; 5 Faculty of Social Sciences, Goethe University Frankfurt, Frankfurt am Main, Germany; 6 Institute for Empirical Analytical Research (InFER), Goethe University Frankfurt, Frankfurt am Main, Germany

**Keywords:** Arabic validation, cross-cultural adaptation, impostor phenomenon, Leary Impostor Scale, psychometric validation

## Abstract

**Background:**

The Leary Impostorism Scale (LIS) is a brief established instrument designed to assess impostorism; however, no validated Arabic version is currently available. This study aimed to translate, culturally adapt, and evaluate the psychometric properties of the Arabic LIS among university students.

**Methods:**

A cross-sectional validation study was conducted among Arabic-speaking university students in Saudi Arabia. Participants completed the Arabic LIS and comparator measures of impostorism, self-efficacy, and perfectionism. Psychometric evaluation included internal consistency, exploratory and confirmatory factor analyses, construct and concurrent validity, and test–retest reliability.

**Results:**

A total of 185 participants with complete LIS data were included in the psychometric analyses. The Arabic LIS demonstrated excellent internal consistency (Cronbach’s *α* = 0.93; McDonald’s *ω* = 0.93). Factorability indices supported factor analysis [KMO = 0.90; Bartlett’s *χ*^2^(21) = 1536.59, *p* < 0.001]. Exploratory factor analysis based on polychoric correlations supported a single-factor structure, with factor loadings ranging from 0.81 to 0.94 and an eigenvalue of 5.45. Confirmatory factor analysis showed statistically significant freely estimated loadings (*p* < 0.001), with standardized loadings ranging from 0.78 to 1.00; global fit indices were *χ*^2^(14) = 105.39, CFI = 0.91, TLI = 0.87, RMSEA = 0.19 (90% CI [0.16, 0.22]), and SRMR = 0.06. Strong positive correlations were observed with the CIPS-20 (*r* = 0.76), CIPS-10 (*r* = 0.77), and IPSS-3 (*r* = 0.66), whereas weaker correlations were found with general self-efficacy (*r* = 0.20) and perfectionism (*r* = 0.16–0.18). Test–retest reliability was moderate among 14 participants retested after 1–4 weeks (Pearson *r* = 0.65; ICC = 0.65).

**Conclusion:**

The Arabic LIS demonstrated high internal consistency and promising initial evidence of validity as a brief measure of impostorism among Arabic-speaking university students. Further evaluation of its factor structure and temporal stability in a broader population is warranted.

## 1 Introduction

The impostor phenomenon (IP) is characterized by persistent feelings of intellectual or professional fraudulence despite objective evidence of competence and achievement. Individuals experiencing the IP often discount their accomplishments, attribute success to external factors such as luck or excessive effort, and fear being exposed as less capable than others believe (Bravata et al., 2020; Huecker et al., 2026). Originally described by Clance and Imes (1978) among high-achieving women, IP has since been documented across genders, educational levels, career stages, and diverse professions. In academic settings, frequent evaluation and comparison may undermine confidence, belonging, help-seeking, and engagement, highlighting the need to study impostorism among students (Alrayyes et al., 2020; Franchi and Russell-Sewell, 2023).

The IP is assessed primarily using self-report instruments, including the Clance Impostor Phenomenon Scale (CIPS), Harvey Impostor Phenomenon Scale (HIPS), Perceived Fraudulence Scale (PFS), and Leary Impostorism Scale (LIS). However, these instruments vary in their conceptualization and psychometric properties, and no single measure has been established as the gold standard for assessing IP (Mak et al., 2019). Developed by Leary et al. (2000), the LIS is a 7-item, 5-point Likert self-report instrument measuring the unidimensional construct of impostorism. Its short length reduces respondent burden and makes it suitable for studies assessing impostorism alongside several educational or psychological variables (Leary et al., 2000).

In medical education, academic performance is an indicator of future professional success. Although the relationship between impostorism and academic performance has been widely investigated, findings remain inconsistent. Students with stronger impostor feelings often report greater perfectionism, academic anxiety, reduced self-efficacy, and avoidance of academic challenges (Clance and Imes, 1978; King and Cooley, 1995; Franchi and Russell-Sewell, 2023; Ménard and Chittle, 2023; Huecker et al., 2026). While several studies have reported that higher impostor scores are associated with poorer examination performance or lower perceived academic competence (Alrayyes et al., 2020; Elnaggar et al., 2023; Franchi and Russell-Sewell, 2023; Diaconescu et al., 2024), others have found weak or no significant relationship with GPA (King and Cooley, 1995). These findings suggest that impostorism may substantially impair students’ educational experiences even when academic achievement is maintained through compensatory effort, highlighting the need for further research across diverse cultural and educational settings (King and Cooley, 1995; Alrayyes et al., 2020; Bravata et al., 2020; Elnaggar et al., 2023; Franchi and Russell-Sewell, 2023; Ménard and Chittle, 2023; Diaconescu et al., 2024).

As outlined by Beaton et al. (2000), translating a psychometric instrument requires more than literal linguistic translation. Cross-cultural adaptation is necessary to achieve semantic, idiomatic, experiential, and conceptual equivalence, while ensuring that the translated instrument measures the same underlying construct as the original. This process should be followed by psychometric evaluation in the target population to establish reliability and validity (Beaton et al., 2000). Although CIPS has been recently validated in Arabic (AbuDujain et al., 2025), to our knowledge, no validated Arabic version of the LIS has been reported. This limits the use of the LIS in Arabic-speaking populations and the comparability of findings with studies using the original instrument. Accordingly, this study aimed to translate and culturally adapt the LIS into Arabic and evaluate its psychometric properties among Arabic-speaking university students.

## 2 Methodology

### 2.1 Study design and setting

This cross-sectional validation study was conducted in Riyadh, Saudi Arabia, from May to July 2026. Students from King Saud University across different colleges, including health and non-health colleges, were recruited for this study. Eligible participants were Arabic-speaking students aged 18 years or older, currently enrolled as regular students, and willing to participate in the study. Exclusion criteria included non-Arabic speakers and inability to provide informed consent.

The sample size was guided by commonly used recommendations for psychometric and factor-analytic studies, including consideration of the number of scale items and model complexity (Wolf et al., 2013). Given that the LIS contains 7 items, a sample exceeding 10 participants per item was sought. A total of 185 participants were ultimately included in the psychometric analyses.

### 2.2 Measures

#### 2.2.1 Leary Impostorism Scale (LIS)

The Leary Impostorism Scale (LIS) is a 7-item self-report instrument developed by Leary et al. to assess impostorism; each item is rated on a 5-point Likert scale from 1 (not at all characteristic) to 5 (extremely characteristic). The scale evaluates the extent to which individuals doubt their abilities, discount or fail to internalize their accomplishments, attribute success to external factors such as luck or effort rather than ability, and fear that others will eventually discover their perceived lack of competence (Leary et al., 2000). The original study reported good internal consistency (Cronbach’s *α* = 0.87) and strong correlations with other impostor measures. Total scores range from 7 to 35, with higher scores indicating greater levels of impostorism (Leary et al., 2000). The Arabic version of the LIS was the primary instrument evaluated in the present study.

#### 2.2.2 Clance Impostor Phenomenon Scale–20 and–10

The Clance Imposter Phenomenon Scale (CIPS) was originally developed as a 20-item self-report instrument (CIPS-20) to assess the severity of the impostor phenomenon, including persistent feelings of fraudulence and self-doubt, fear of being exposed as incompetent, difficulty internalizing success, and the tendency to attribute success to external factors rather than one’s own ability (Chrisman et al., 1995). The Clance Imposter Phenomenon Scale-10 (CIPS-10) is a brief version of the original instrument that measures the same concept while reducing respondent burden and maintaining good psychometric performance (Wang et al., 2024). The Arabic version of the CIPS has demonstrated strong internal consistency (Cronbach’s *α* = 0.90) and good construct validity in an Arabic-speaking population (AbuDujain et al., 2025; Alsaad and AbuDujain, 2026).

#### 2.2.3 Impostor Phenomenon Short Scale (IPSS-3)

The Impostor Phenomenon Short Scale (IPSS-3) is a brief three-item self-report instrument developed to assess IP intensity while reducing respondent burden. It provides a concise measure of the overall severity of IP experiences, with higher scores indicating greater IP intensity (Jansen, 2024). The IPSS-3 has been cross-nationally validated and represents a unique, brief instrument with remarkable psychometric quality (Jansen and Yaffe, 2025). The IPSS-3 allows for capturing IP intensity in large-scale, longitudinal surveys (Jansen et al., under review)^1^.

#### 2.2.4 General Self-Efficacy Scale (GSES)

The General Self-Efficacy Scale (GSES) is a 10-item self-report instrument developed to assess an individual’s general belief in their ability to cope with challenging situations and successfully manage a wide range of demanding circumstances. Items are rated on a 4-point Likert scale ranging from 1 (not at all true) to 4 (exactly true), with higher scores indicating greater perceived self-efficacy, which is generally associated with lower levels of impostor phenomenon. The original scale demonstrated good internal consistency across multiple populations (Schwarzer and Jerusalem, 1995; Crandall et al., 2016).

#### 2.2.5 Big Three Perfectionism Scale-Short Form (BTPS-SF)

The Big Three Perfectionism Scale-Short Form (BTPS-SF) is a 16-item self-report instrument designed to assess three high-order dimensions of perfectionism: rigid perfectionism, self-critical perfectionism, and narcissistic perfectionism. Items are rated on a 5-point Likert scale, with higher scores indicating greater levels of perfectionism across these three dimensions (Smith et al., 2016; Feher et al., 2020; Fekih-Romdhane et al., 2023).

### 2.3 Translation and cultural adaptation

The translation and cross-cultural adaptation process followed established international guidelines (Beaton et al., 2000; Sousa and Rojjanasrirat, 2011). Initially, two independent forward translations into Arabic were performed, after which the translated versions were reviewed and harmonized by NMA into a single preliminary version. This version was then independently back-translated into English. As the original scale developer could not be reached, the back-translated version was reviewed by two native English speakers from the United Kingdom to assess its consistency with the original instrument and identify any discrepancies in meaning. All identified issues were discussed and resolved. The resulting Arabic version subsequently underwent face-validity assessment, and feedback from this stage was incorporated into the instrument. Following these revisions, the final Arabic version was produced and used for psychometric testing (Appendix 1).

### 2.4 Data collection and variables

Data were collected using an electronic, self-administered questionnaire administered via SurveyMonkey. The first page of the survey provided study details including study objectives, voluntary participation, and confidentiality. The questionnaire, which took approximately 10 min to complete, collected demographic information, the Arabic version of the Leary Impostorism Scale (LIS), and additional validated instruments to assess construct validity. To assess test–retest reliability, 14 participants completed the Arabic LIS again 1–4 weeks after the initial assessment.

### 2.5 Ethical considerations

This study was approved by the Institutional Review Board (IRB) of the College of Medicine, King Saud University in May 2026 (Ref. No R26-IRB-245). All participants provided electronic informed consent before beginning the survey.

### 2.6 Statistical analysis

All psychometric analyses were conducted using Stata 16 on participants with complete responses to all seven LIS items (*N* = 185); analyses involving the GSES and BTPS-SF dimensions were based on the available subsample of 81 participants. Before conducting the analyses, the dataset was prepared by creating all composite scale scores and restricting the analytic sample to respondents with complete responses on the LIS items. The validation strategy followed standard recommendations for scale validation and included descriptive statistics, internal consistency analyses, exploratory and confirmatory factor analyses, construct and concurrent validity analyses, and detailed item analyses. Internal consistency was evaluated using Cronbach’s *α*, McDonald’s *ω*, corrected item–total correlations, and *α* if item deleted.

Since the LIS consists of seven ordinal Likert-type items (1–5), analyses were adapted accordingly. Factorability was assessed using the Kaiser–Meyer–Olkin (KMO) measure and Bartlett’s test of sphericity. Exploratory factor analysis was conducted using a polychoric correlation matrix with principal factor extraction, and factor retention was evaluated using eigenvalues and inspection of the scree plot. Both the EFA and CFA were conducted using the same complete-case sample (*N* = 185). Confirmatory factor analysis evaluated the hypothesized one-factor structure of the LIS using maximum likelihood (ML) estimation. Model fit was assessed using the *χ*^2^ test, comparative fit index (CFI), Tucker–Lewis index (TLI), root mean square error of approximation (RMSEA) with 90% confidence intervals, and standardized root mean square residual (SRMR).

Construct and concurrent validity were assessed using Pearson correlations between LIS scores and the CIPS-20, CIPS-10, IPSS-3, GSES, and BTPS-SF dimensions, with 95% confidence intervals. Test–retest reliability was evaluated using Pearson correlation and an intraclass correlation coefficient (ICC) estimated using a two-way mixed-effects model for absolute agreement. Agreement between baseline and retest scores was additionally examined using Bland–Altman analysis.

## 3 Results

The psychometric evaluation of the Arabic LIS was conducted using a complete-case sample of 185 respondents who provided valid responses on all 7 items. Descriptive statistics are presented in Table 1. Most participants were medical students (143/185, 77.3%), while only 11 (5.9%) were enrolled in non-health specialties. The overall mean LIS item score was 1.85 (SD = 0.95) on the 1–5 response scale. Female respondents had slightly higher mean scores than males (1.92 vs. 1.72). Mean scores according to academic major are also presented.

**Table 1:** Descriptive statistics (*N* = 185).

Variable		*N*	Mean	SD	Min	Max
Gender	Male	65	1.72	0.82	1.00	4.00
	Female	120	1.92	1.01	1.00	5.00
Major	Medicine	143	1.78	0.87	1.00	5.00
	Nursing	19	2.32	1.28	1.00	4.86
	Pharmacy	1	1.14	.	1.14	1.14
	Allied medical	11	2.25	1.29	1.00	5.00
	Non-health specialty	11	1.65	0.70	1.00	3.00

Reliability results are summarized in Table 2, while factorability and factor analysis results are presented in Table 3. The Arabic LIS demonstrated excellent internal consistency, with Cronbach’s α and McDonald’s ω both equal to 0.93 and a mean inter-item correlation of 0.67. Test–retest reliability yielded a Pearson correlation of 0.65 and an intraclass correlation coefficient (ICC) of 0.65. The data were suitable for factor analysis (KMO = 0.90; Bartlett’s *χ*^2^(21) = 1536.59, *p* < 0.001). Exploratory factor analysis based on polychoric correlations supported a single-factor structure, with factor loadings ranging from 0.81 to 0.94 and a first-factor eigenvalue of 5.45. The scree plot demonstrated a clear break after the first factor (Figure 1), further supporting a one-factor solution. The polychoric correlation matrix (Supplementary Table S1) showed positive correlations among all seven items.

**Table 2:** Reliability statistics.

Reliability statistic	Cronbach’s *α*	McDonald’s *ω*	Pearson *r*	ICC
Arabic LIS	0.93	0.93	0.65	0.65

α, Cronbach’s alpha; ω, McDonald’s omega; ICC, intraclass correlation coefficient. Pearson r and ICC were calculated using the test–retest subsample. ICC was estimated using a two-way mixed-effects model for absolute agreement.

**Table 3:** Item-level descriptive statistics, item analysis, and factor loadings of the Arabic LIS (*N* = 185).

Item	*M* (SD)	Skew.	Kurt.ᵃ	Floor, %ᵇ	Corrected item–total *r*	α if deleted	EFA loadingᶜ	CFA loading (SE)ᵈ	CFA std. loadingᵈ
LIS1	1.69 (1.00)	1.41	4.31	60.00	0.82	0.91	0.91	1.00 (0.00)	0.84
LIS2	1.74 (1.08)	1.42	4.17	58.92	0.85	0.91	0.94	1.17 (0.07)	0.97
LIS3	2.23 (1.29)	0.68	2.26	41.08	0.75	0.92	0.85	1.14 (0.10)	0.96
LIS4	1.65 (1.03)	1.56	4.66	63.78	0.81	0.91	0.91	1.05 (0.07)	0.88
LIS5	2.36 (1.36)	0.57	2.01	37.84	0.70	0.93	0.81	1.14 (0.10)	0.95
LIS6	1.75 (1.13)	1.57	4.61	58.92	0.82	0.91	0.91	1.20 (0.08)	1.00
LIS7	1.53 (1.02)	1.96	5.99	73.51	0.71	0.92	0.85	0.93 (0.08)	0.78

LIS, Leary Impostorism Scale; EFA/CFA, exploratory/confirmatory factor analysis; SE, standard error.

ᵃKurtosis values are raw; under the raw convention a normal distribution = 3.

ᵇPercentage of respondents selecting the lowest response option (1, “not at all characteristic”).

ᶜSingle-factor EFA on polychoric correlations, principal factor extraction. Factorability: KMO = 0.90; Bartlett’s *χ*^2^(21) = 1,536.59, *p* < 0.001; eigenvalue = 5.45, accounting for 96.58% of the estimated common variance in the principal-factor solution.

ᵈOne-factor CFA, maximum likelihood estimation; LIS1 fixed at 1.00 for identification; all freely estimated loadings *p* < 0.001. Model fit: *χ*^2^(14) = 105.39, CFI = 0.91, TLI = 0.87, RMSEA = 0.19 (90% CI 0.16–0.22), SRMR = 0.06.

**Figure 1:**
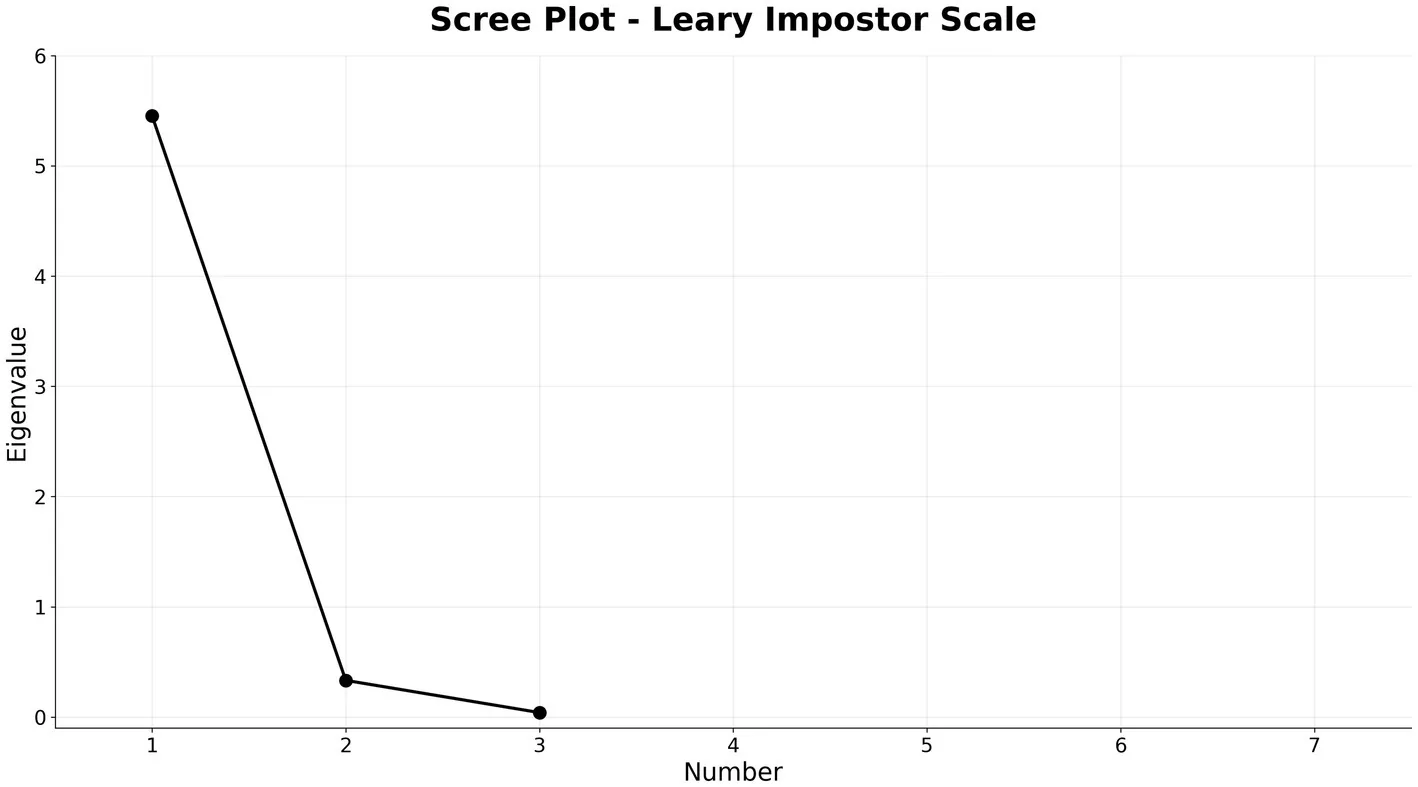
Scree plot of eigenvalues from the exploratory factor analysis of the Arabic LIS, showing a clear elbow after the first component, consistent with a one-factor structure.

The exploratory and confirmatory factor analysis results are presented in Table 3, and the corresponding one-factor CFA model is illustrated in Figure 2. Exploratory factor analysis based on polychoric correlations yielded factor loadings ranging from 0.81 to 0.94. The first factor had an eigenvalue of 5.45 and accounted for 96.58% of the estimated common variance in the principal-factor solution. The polychoric correlation matrix (Supplementary Table S1) showed positive correlations among all seven items. Confirmatory factor analysis demonstrated that all freely estimated factor loadings were statistically significant (*p* < 0.001), with standardized factor loadings ranging from 0.78 to 1.00. Global fit indices for the single-factor CFA were χ^2^(14) = 105.39, *p* < 0.001, CFI = 0.91, TLI = 0.87, RMSEA = 0.19 (90% CI [0.16, 0.22]), and SRMR = 0.06. The fit indices were mixed: the CFI and SRMR provided some support for the model, whereas the TLI and particularly the RMSEA indicated suboptimal fit. Given the elevated RMSEA and TLI below conventional thresholds, the CFA findings should be interpreted cautiously despite the strong standardized factor loadings.

**Figure 2:**
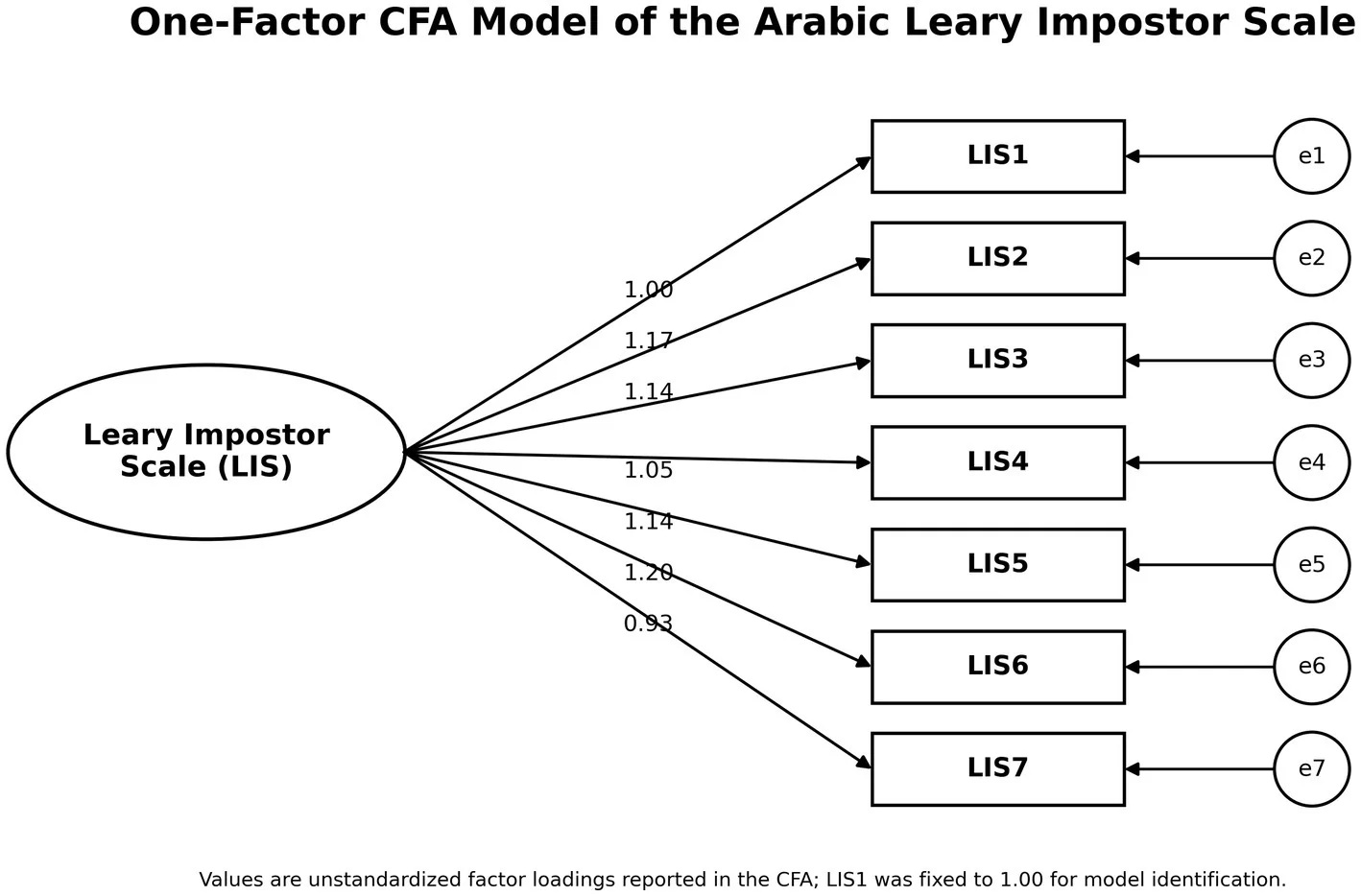
Confirmatory factor analysis of the Arabic Leary Impostor Scale (LIS). One-factor CFA model of the seven-item Arabic LIS. Rectangles represent observed items, the ellipse represents the latent construct, circles represent error terms, and values on the arrows indicate unstandardized factor loadings.

Construct and concurrent validity results are presented in Table 4. The Arabic LIS showed strong positive correlations with the CIPS-20 (*r* = 0.76), CIPS-10 (*r* = 0.77), and IPSS-3 (*r* = 0.66). Smaller positive correlations were observed with general self-efficacy (*r* = 0.20) and the three perfectionism dimensions of the BTPS (*r* = 0.16–0.18). All reported correlations were statistically significant.

**Table 4:** Construct and concurrent validity of the Arabic LIS (*N* = 185).

Measure	Pearson *r*	95% CI	*p*-value
CIPS 20	0.76	(0.69–0.81)	<0.001
CIPS 10	0.77	(0.70–0.82)	<0.001
IPSS 3	0.66	(0.57–0.73)	<0.001
GSES	0.20	(0.05–0.33)	<0.001
BTPS – Rigid	0.16	(0.01–0.29)	0.03
BTPS – SelfCritical	0.18	(0.03–0.31)	0.02
BTPS – Narcissistic	0.18	(0.03–0.31)	0.02

BTPS, Big Three Perfectionism Scale; CIPS-20, 20-item Clance Impostor Phenomenon Scale; CIPS-10, 10-item Clance Impostor Phenomenon Scale; CI, confidence interval; GSES, General Self-Efficacy Scale; IPSS-3, 3-item Impostor Phenomenon Short Scale. Pearson r coefficients represent bivariate correlations between the Arabic LIS and comparator measures. *N* = 185.

Test–retest reliability results are summarized in Table 2, while agreement between baseline and retest scores is illustrated in the Bland–Altman plot (Figure 3). The mean difference between measurements was 0.05, with limits of agreement ranging from −1.21 to 1.32.

**Figure 3:**
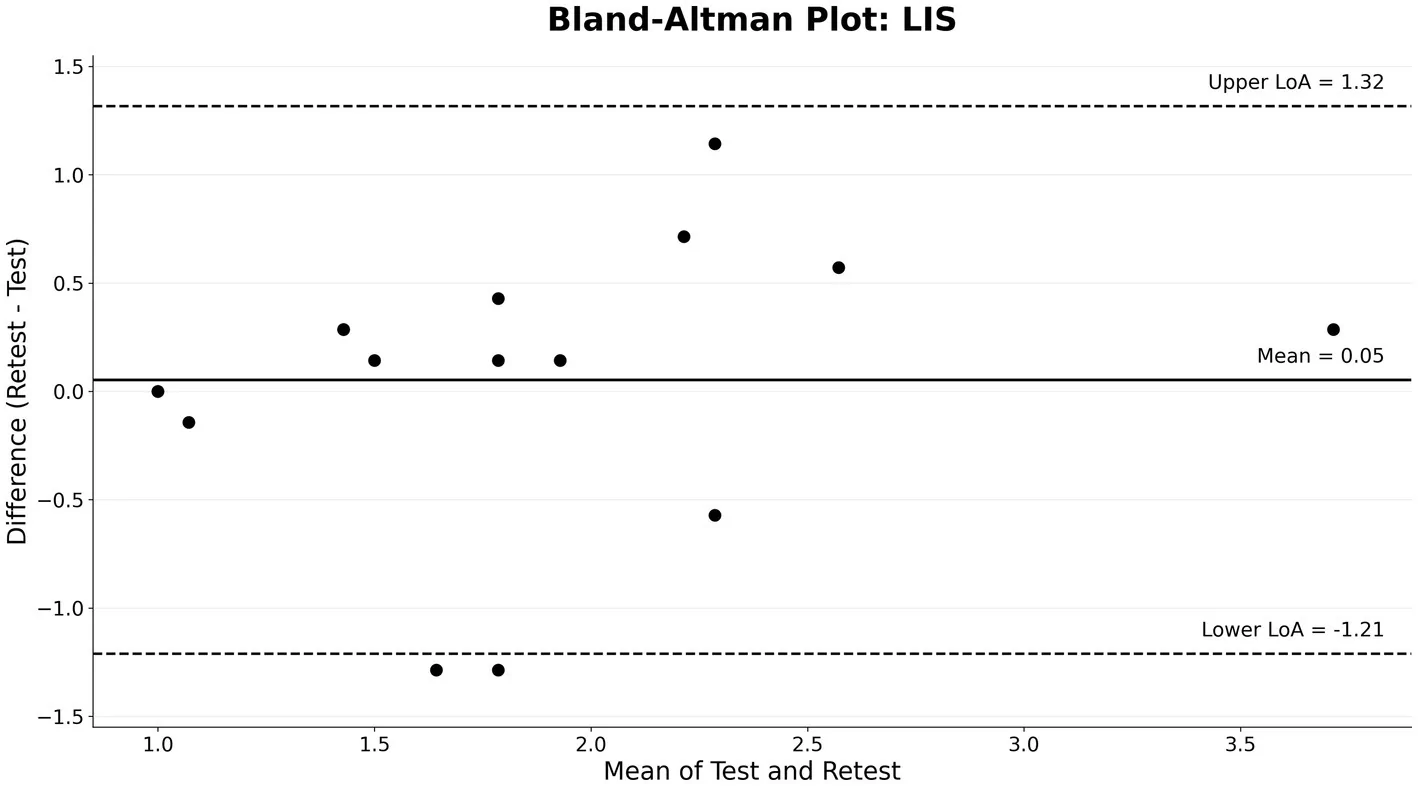
Bland–Altman plot for test–retest agreement of the Arabic Leary Impostor Scale (LIS). The solid line represents the mean difference (bias) between test and retest scores, while the dashed lines indicate the 95% limits of agreement (−1.21 to 1.32). Each point represents an individual participant.

## 4 Discussion

Impostorism describes a persistent sense of intellectual fraudulence that survives objective evidence of competence (Clance and Imes, 1978; Bravata et al., 2020). It is common in higher education and the health professions, where frequent evaluation and peer comparison give self-doubt repeated occasions to surface (Bravata et al., 2020; Franchi and Russell-Sewell, 2023). Impostor feelings have been linked to burnout, anxiety, depressive symptoms, and reduced self-esteem across training settings (Alrayyes et al., 2020; Franchi and Russell-Sewell, 2023; Diaconescu et al., 2024; Huecker et al., 2026), and Saudi student and trainee samples reproduce this pattern (Alrayyes et al., 2020; Elnaggar et al., 2023). Measurement has not kept pace with that literature. Several self-report instruments compete for use; none has been accepted as a criterion standard, and a systematic appraisal concluded that the supporting evidence remains uneven (Mak et al., 2019). This study translated the seven-item LIS into Arabic and evaluated its internal consistency, temporal stability, factor structure, and relationships with established measures of impostorism, self-efficacy, and perfectionism.

The mean Arabic LIS score was 1.85 (SD = 0.95) on a five-point metric, placing this sample toward the lower end of the response range. Women scored slightly higher than men (1.92 versus 1.72), a descriptive difference only, since measurement invariance across gender was not tested. Gender effects have in any case been inconsistently observed across the impostorism literature (Ménard and Chittle, 2023), and Leary et al. (2000) reported virtually identical scores for male and female respondents on the original scale. Two prior LIS samples offer a reference point. Martin et al. (2024) reported item means between 2.12 and 2.94 in Australian postgraduate psychology trainees, and Freeman et al. (2022) reported item means between 1.74 and 2.47 in healthcare simulation educators. The present sample sits at or below the lower of those ranges. A developmental reading is tempting, in which impostorism deepens as trainees take on independent responsibility, but the available evidence does not support it. Several studies collected in a review of post-secondary populations found impostorism unrelated to year of training or to residency year (Ménard and Chittle, 2023). The three samples also differ in nationality, discipline, career stage, and language of administration (Freeman et al., 2022; Martin et al., 2024), and nothing in this design isolates any one of those differences from the others. Whether reporting of self-doubt is attenuated in a setting where acknowledging professional inadequacy carries social cost is a further possibility, but one that no evidence cited here establishes and that the present design cannot test. These comparisons therefore locate the Arabic LIS distribution within the existing literature rather than explaining it, and the same restraint applies to the cross-sample comparisons that follow.

Internal consistency was high, with Cronbach’s alpha and McDonald’s omega both at 0.93 and a mean inter-item correlation of 0.67. This sits within the range established by earlier work: approximately 0.87 in Leary’s undergraduate sample (Leary et al., 2000), 0.94 in Australian postgraduate trainees (Martin et al., 2024), 0.94 in healthcare simulation educators (Freeman et al., 2022), and above 0.90 in both Brazilian Portuguese samples (Silva et al., 2026). Values of this order have been reported across different languages and populations, suggesting broadly consistent internal consistency across the available LIS studies. The high inter-item correlation does raise a question of redundancy, and Martin et al. (2024) identified the same feature as grounds for testing further item reduction. Temporal stability was weaker. The Pearson coefficient and the ICC were both 0.65, moderate under conventional criteria (Koo and Li, 2016) and below the 0.70 threshold applied to reproducibility in health status instruments (Terwee et al., 2007). Bland–Altman analysis showed negligible systematic drift (mean difference 0.05), though limits of agreement from −1.21 to 1.32 were wide against a five-point metric. However, these estimates were based on only 14 participants and should be interpreted cautiously. A larger retest sample with a prespecified interval is needed to assess temporal stability more precisely. Neither the original scale nor the Australian and simulation-educator validations reported retest data, and Martin et al. (2024) identified its assessment as a requirement for future work, so this estimate stands without a prior LIS benchmark. The factor-analytic findings should be considered in relation to the original scale development. Leary et al. (2000) constructed the LIS as a unidimensional measure drawn from items in existing multidimensional instruments and reported internal consistency and concurrent validity, but they did not report a formal exploratory or confirmatory factor analysis of the seven items, and a systematic review of impostorism measures later noted that the available instruments had yet to undergo systematic evaluation of their psychometric properties (Mak et al., 2019). Structural evidence accumulated only afterwards. Freeman et al. (2022) published the first exploratory analysis and recovered a single factor explaining 73.4% of the variance, Martin et al. (2024) obtained one factor explaining 73.67%, and the Brazilian adaptation paired exploratory analysis in one sample with confirmatory analysis in a second (Silva et al., 2026). The present findings extend that sequence. Factorability was adequate (KMO = 0.90; Bartlett’s *χ*^2^(21) = 1536.59, *p* < 0.001), and the polychoric EFA supported a single-factor structure, with an eigenvalue of 5.45 and factor loadings ranging from 0.81 to 0.94. CFA likewise showed strong standardized factor loadings (0.78–1.00), although the global fit indices were mixed. The CFI (0.91) and SRMR (0.06) provided some support for the one-factor model, whereas the TLI (0.87) and particularly the RMSEA (0.19, 90% CI [0.16, 0.22]) indicated suboptimal fit. Given the elevated RMSEA and TLI below conventional thresholds, the CFA findings should be interpreted cautiously despite the strong standardized factor loadings. The exploratory analysis used polychoric correlations to account for the ordinal response format of the LIS items (Flora and Curran, 2004; Terwee et al., 2007). To our knowledge, this is the first factor-analytic evaluation of the LIS in an Arabic-speaking population.

The validity evidence was mixed. Against dedicated impostorism measures, the correlations were strong to moderate: 0.76 with the CIPS-20, 0.77 with the CIPS-10, and 0.66 with the IPSS-3 (Jansen, 2024; Wang et al., 2024; AbuDujain et al., 2025; Alsaad and AbuDujain, 2026). Leary et al. (2000) reported correlations of 0.70 to 0.80 between the original scale and three impostorism measures available in 2000, one of which was an earlier version of the Clance scale. The CIPS-10 and IPSS-3 are more recent derivatives that did not exist at that time, so this range offers a loose historical benchmark rather than a like-for-like comparison; against it, the CIPS-20 and CIPS-10 coefficients sit close to or within range, while the IPSS-3 coefficient sits below it. The Brazilian adaptation likewise found a positive association between the LIS and the Clance scale, and agreement extends that far (Silva et al., 2026). It does not extend to perfectionism. That study reported a positive association between LIS scores and maladaptive perfectionism (Silva et al., 2026), whereas correlations here with all three BTPS dimensions were weak and closely clustered, between 0.16 and 0.18 (Smith et al., 2016). The clearest differences emerged in comparison with the Arabic CIPS validation. That validation, conducted in a comparable Saudi population, modeled impostorism as a higher-order construct and reported a path coefficient of −0.39 to general self-efficacy, measured with the GSES (Schwarzer and Jerusalem, 1995), together with path coefficients of 0.46 to rigid perfectionism and 0.68 to self-critical perfectionism (AbuDujain et al., 2025). The Arabic LIS produced a weak positive correlation with self-efficacy (*r* = 0.20) and uniformly weak correlations with perfectionism. Three considerations bear on the divergence. The first is metric and precision: those estimates are path coefficients from a structural model rather than bivariate correlations (AbuDujain et al., 2025), and the present comparisons rested on a subsample of 81 respondents, which widens the uncertainty around every coefficient reported here. The second is range: a sample scoring near the floor of the LIS offers limited variance for detecting linear association. The third is item content, and it is the most consequential. The CIPS covers substantially broader ground. Its factor structure has been described as comprising luck, fake, and discount components (Freeman et al., 2022; Martin et al., 2024), and its items include dread of being evaluated and disappointment at falling short of one’s own standards (Freeman et al., 2022), content that overlaps directly with perfectionism and with beliefs about personal capability. Whether those components constitute genuine subscales remains contested, with several analyses favoring a single factor (Mak et al., 2019; Freeman et al., 2022; Martin et al., 2024), but the breadth of the item pool is not in dispute. The LIS is built differently. Its seven items address the sense of being a phony and the fear of exposure, with no reference to attributional style or to the setting of standards (Leary et al., 2000). Leary et al. (2000) deliberately excluded items assessing empirical correlates rather than defining features of impostorism when constructing the scale, so attenuated associations follow from its stated design. That argument covers magnitude but not direction, and the two comparisons therefore stand differently. The perfectionism correlations are weak but run in the positive direction reported by both comparison studies (AbuDujain et al., 2025; Silva et al., 2026), and the narrower content of the LIS may partly account for their magnitude; these weak positive associations are consistent with discriminant validity, although this interpretation was not specified *a priori*. The weak positive association with general self-efficacy (*r* = 0.20) was contrary to the expected negative relationship, as reported in the Arabic CIPS (AbuDujain et al., 2025). This finding should therefore not be interpreted as evidence supporting construct or discriminant validity. The reduced sample size and difference in item content may contribute to the discrepancy, but these explanations remain untested. Replication in a larger sample is needed to clarify this relationship. These convergent and discriminant expectations were not specified before analysis, and the item-content account is offered after the fact rather than as a tested prediction. The practical consequence is that the two scales are not interchangeable. The CIPS samples a wider range of impostorism-related content across 20 items, although in research and applied settings it is generally scored as a single total rather than as subscales (Mak et al., 2019). The Arabic LIS captures the core experience in seven (Leary et al., 2000), which suits studies in which impostorism is one variable among many and respondent burden matters (Martin et al., 2024).

Several features strengthen confidence in these findings. The exploratory analysis used polychoric correlations to account for the ordinal item format, while confirmatory factor analysis was estimated using maximum likelihood. Validity was examined against three impostorism instruments of differing lengths, alongside measures of self-efficacy and perfectionism, so that convergent and discriminant patterns could both be read from the same dataset, although the discriminant comparisons were interpreted after the fact rather than predicted in advance. Temporal stability was evaluated with both a correlation coefficient and an agreement analysis. Limitations qualify what follows from them. The design was cross-sectional and responsiveness to change was not assessed, so the scale cannot yet be recommended for tracking intervention effects. Participants were recruited from a single Saudi university, and 77.3% were medical students. The findings therefore apply most directly to the population studied, with limited generalizability to non-health disciplines and other Arabic-speaking settings. Uneven distribution across academic majors precluded subgroup comparisons, and the self-efficacy and perfectionism correlations were based on a reduced subsample, which widened the intervals around those estimates. Measurement invariance across gender was not tested, so the observed difference between male and female scores cannot be interpreted as a difference in the underlying construct. No criterion standard for impostorism exists, so concurrent validity could be established only against self-report instruments that share the same method variance and its attendant social desirability bias. Although the overall sample exceeded commonly used participant-to-item recommendations, N = 185 remains modest for precise estimation of smaller correlations. In addition, the EFA and CFA were conducted on the same sample; consequently, the CFA does not provide an independent cross-validation of the factor structure. Splitting the available sample would have substantially reduced the precision of both analyses, but independent replication in a larger sample remains necessary. These findings should therefore be regarded as initial psychometric evidence requiring replication in larger samples. Two studies would address the principal gaps. A multi-center study recruiting from at least three Arabic-speaking countries, with balanced enrolment across health and non-health faculties, would permit tests of measurement invariance by country, gender, and discipline, and would establish whether the score distribution observed here reflects the population or the sampling frame. A prospective cohort administering the Arabic LIS at the start and end of a clinical rotation, with a specified retest interval and with examination performance and burnout as outcomes, could help evaluate responsiveness, longitudinal validity, and predictive utility.

## 5 Conclusion

The Arabic Leary Impostorism Scale demonstrated high internal consistency, high enough to raise a question of item redundancy worth testing directly, and a predominantly one-factor structure supported by the exploratory analysis and strong CFA factor loadings, although global CFA fit indices were mixed. Convergent validity was strong to moderate across three established impostorism instruments, with the weakest below the historical benchmark reported for the original scale. Its correlations with perfectionism were weak and ran in the direction reported by comparison studies, consistent with, but not definitive evidence of, discriminant validity; its weak, positively signed correlation with general self-efficacy departs from the negative association reported for the Arabic CIPS and is better read as an open question than as evidence of discriminant validity. Temporal stability was moderate, but it should be interpreted with caution because only 14 participants completed the retest. These results support the Arabic LIS as a promising brief measure of impostorism for Arabic-speaking university students, suited to survey research in which impostorism is one of several constructs under study, though its factor structure, temporal stability, and relationship to self-efficacy warrant further examination before the instrument is treated as fully established. Confirmation in broader Arabic-speaking samples, together with evaluation of responsiveness over time, would extend its application from research settings into educational monitoring.

## Data Availability

The raw data supporting the conclusions of this article will be made available by the authors, without undue reservation.

## Footnotes

^1^Jansen, M. P., AbuDujain, N. M., Muqresh, M. A., Alfaqir, O. H., and Alsaad, S. M. (under review). Arabic Version of Impostor Phenomenon Short Scale (Ar-IPSS-3): Development, Validation and Cultural Adaptation in Saudi Arabia.
