# Supplementary material for: The Arabic Leary Impostorism Scale: initial psychometric evaluation in a university student population
Source: Front Psychol. 2026 Oct 21;17:1973835. doi: 10.3389/fpsyg.2026.1973835 (PMC13731288; doi:10.3389/fpsyg.2026.1973835)
Supplement: Supplementary file 1 [file Table_1.docx]

**Supplementary Material**

**Table S1**. Polychoric correlation matrix

| **Item** | **LIS 1** | **LIS 2** | **LIS 3** | **LIS 4** | **LIS 5** | **LIS 6** | **LIS 7** |
| --- | --- | --- | --- | --- | --- | --- | --- |
| LIS 1 | 1.00 | 0.77 | 0.74 | 0.72 | 0.65 | 0.68 | 0.59 |
| LIS 2 | 0.77 | 1.00 | 0.65 | 0.75 | 0.61 | 0.84 | 0.69 |
| LIS 3 | 0.74 | 0.65 | 1.00 | 0.61 | 0.73 | 0.58 | 0.52 |
| LIS 4 | 0.72 | 0.75 | 0.61 | 1.00 | 0.57 | 0.78 | 0.71 |
| LIS 5 | 0.65 | 0.61 | 0.73 | 0.57 | 1.00 | 0.59 | 0.46 |
| LIS 6 | 0.68 | 0.84 | 0.58 | 0.78 | 0.59 | 1.00 | 0.73 |
| LIS 7 | 0.59 | 0.69 | 0.52 | 0.71 | 0.46 | 0.73 | 1.00 |
